# The Predatory Bacteria *Bdellovibrio bacteriovorus* LR3: A Potential Biocontrol Agent Against Gram-Negative Pathogenic Microorganisms

**DOI:** 10.3390/microorganisms14010190

**Published:** 2026-01-15

**Authors:** Anna P. Shorokhova, Valentina N. Polivtseva, Tatiana N. Abashina, Vladimir V. Sorokin, Alexey V. Chekanov, Alexander S. Reshetnikov, Alexander G. Bogun, Yanina A. Delegan, Andrei A. Zimin, Nataliya E. Suzina

**Affiliations:** 1 G.K. Skryabin Institute of Biochemistry and Physiology of Microorganisms, Federal Research Center Pushchino Scientific Center for Biological Research, Russian Academy of Sciences, 142290 Pushchino, Russia; 2 Winogradsky Institute of Microbiology, Federal Research Center of Biotechnology, Russian Academy of Sciences, 117312 Moscow, Russia; 3 Institute of Theoretical and Experimental Biophysics, Russian Academy of Sciences, 142290 Pushchino, Russia; 4 D.I. Ivanovsky’s Academy of Biology and Biotechnology, Southern Federal University, 344006 Rostov-on-Don, Russia

**Keywords:** *Bdellovibrio bacteriovorus*, *Pseudomonas tolaasii*, cultivated mushrooms, *Agaricus bisporus*

## Abstract

The paper describes a predatory Gram-negative bacterium from the genus *Bdellovibrio*, which was isolated from water of the Lyubozhikha River. As revealed by electron microscopy, the bacterium is an intracellular predator of Gram-negative microorganisms. Its prey range includes *Pseudomonas tolaasii*, the phytopathogen responsible for brown spot disease in the cultivated button mushroom (*Agaricus bisporus*). Based on the results of a *16S rRNA* gene sequence analysis, the bacterium was identified as *Bdellovibrio bacteriovorus* strain LR3. We characterized the predator–prey dynamics between *B. bacteriovorus* LR3 and *P. tolaasii*, determining the optimal temperature and pH conditions for this interaction. Our results demonstrate the potential of *B. bacteriovorus* LR3 as a biocontrol agent against *P. tolaasii* in mushroom cultivation. The possibility of using *B. bacteriovorus* LR3 against clinical cases *Salmonella* and *Escherichia* infections is also addressed.

## 1. Introduction

Currently, the treatment of infections in humans, animals, plants, and fungi caused by Gram-negative bacteria requires the development of new approaches and methods. This need is driven not only by the high resistance of pathogenic Gram-negative bacteria to existing antibiotics, but also by the limitations and regulatory restrictions on the use of current antimicrobial agents in agriculture [1,2]. One possible approach to combat bacterial infections in humans, animals, and plants is based on the use strains of the predatory bacterium *Bdellovibrio bacteriovorus* [3,4], which can be regarded as “alive antibiotics” [5]. Bdellovibrios are able to penetrate into Gram-negative cells and kill them without harming the surrounding eukaryotic cells [6]. A particular area, where bdellovibrios can be useful, is fungiculture. On farms cultivating edible mushrooms, such as *Agaricus bisporus*, significant economic damage is caused by *Pseudomonas tolaasii* [7]. This pathogenic bacterium infects mushrooms and slows down their growth, which leads to the loss of the harvested biomass [8]. The disease manifests itself as brown lesions in the outer layer of mushroom caps and stems. These brown spots result from the synthesis of melanin by fungal cells, which is a protective response to the toxin tolaasin produced by *P. tolaasii.* Tolaasin destroys the plasma membrane of fungal cells, providing access of *P. tolaasii* to the cell nutrients [9]. As a result, mushrooms lose their visual appeal, which greatly affects the purchasing priorities of the consumers.

In order to combat phytopathogenic microflora and avoid harvest losses, mushroom farms mostly use sanitary measures, such as the chlorination of irrigation water [10] and monitoring of air humidity, which is necessary to control the rapid spread of the pathogen in the soil and on mushroom surfaces due to chemotaxis [11,12] and the high mobility of *P. tolaasii* [6]. More drastic measures for the treatment of mushroom spot include the use of chemical disinfectants, antibiotics [13], natural plant extracts [14], and bacteriophages specific for *P. tolaasii* [15]. Pseudomonad antagonists of *P. tolaasii*, such as *Pseudomonas flourescens*, have also been tested as biocontrol strains [16]. Recent studies support the use of novel bacterial isolates to control this disease [17,18]. The introduction of bdellovibrions decreases the infection symptoms, and the additional treatment of mushroom caps leads to a better preservation of harvest [19].

However, despite being efficacious under laboratory conditions, some of the above-listed approaches are ill-suited for fungiculture. For example, antibiotics and aseptics cannot be used in mushroom farming and, in addition, *P. tolaasii* quickly adapts and becomes resistant to phages, which reduces the efficacy of this option and limits its use for the treatment of cultivated mushrooms. One of the remaining prospective options for the treatment of *P. tolaasii*-infected fungi could, therefore, be *Bdellovibrio* bacteria, which are not toxic to humans, animals and plants [20]. Unfortunately, most predatory bacteria target not only *P. tolaasii* but also *Pseudomonas putida*—commensal symbionts of *Agaricus bisporus*, the presence of which in the soil is necessary to stimulate the initial stages of fungal growth [21,22].

Thus, the objective of this work was to find a strain of *Bdellovibrio* bacteria that infects *P. tolaasii*, but that is not deleterious to *P. putida.*

## 2. Materials and Methods

### 2.1. Isolation and Cultivation of Bacteria

Predatory microorganisms were isolated from the pond water collected at the dam of the Lyubozhikha River near Pushchino (Moscow Region). The isolation was performed using methods developed for *Bdellovibrio* bacteria [3]. A new strain of *Bdellovibrio* was isolated by direct inoculation onto a lawn of prey bacteria [23]. The prey bacteria (*Pseudomonas tolaasii*; a strain from the laboratory collection) were cultivated on a tryptone–soy medium 5/5 of the following composition (g/L): soy extract (AppliChem, Darmstadt, Germany), 30.0; casein hydrolysate (Sigma-Aldrich, St. Louis, MO, USA), 5.0; yeast extract (AppliChem, Darmstadt, Germany), 1.0; aminopeptide (Diam, Moscow, Russia) (ml/L), 60.0; agar (Sigma-Aldrich, St. Louis, MO, USA), 20.0; pH 8.0. The cells were washed off the agar medium with sterile tap water and resuspended in a co-culture (predator–prey binary culture) medium to a concentration of 10^7^–10^8^ CFU(PFU)/mL. Water samples were collected in sterile plastic vials (Rybinsk Instrument Making Plant, Rybinsk, Russia) and processed 2–3 h after sampling. The lytic-spot material was collected after 3, 4, and 5 days of cultivation, and after examination under a phase-contrast microscope, transferred to a suspension of prey cells in tap water (10^7^–10^8^ CFU/mL). After cultivation at 30 °C to the point of medium clarification, the co-culture suspension was filtered through membrane filters with a pore diameter of 0.45 μm (ALWSCI Technologies, Shaoxing, Zhejiang, China). The filtered suspension (0.1 mL) was added to a fresh prey-cell suspension and cultivated again under the same conditions. After completion of the lysis process, the co-culture was filtered through 0.45-μm filters at least thrice.

### 2.2. Predator-Prey Interaction

The interaction in the co-culture of predator and prey was carried out in sterile tap water on a rotary shaker (Biosan, Riga, Latvia (230 rpm)) at 30 °C. The concentration of *Bdellovibrio* cells was determined by the number of lytic spots (Plaque Forming Unit (PFU/mL)) on the lawn of prey cells from the corresponding dilutions using the two-layer agar method. The predation process in the predator–prey co-culture was monitored by changes in the optical density at 600 nm (OD_600_) using a Spekol-211 spectrophotometer (Carl Zeiss Industrielle Messtechnik GmbH, Oberkochen, Germany), as well as by counting the predator (PFU/mL) and prey (CFU/mL) cells from the corresponding dilutions.

### 2.3. Temperature Effect

The effect of temperature (5–45 °C) on the ability of the isolated *Bdellovibrio* strain to prey upon *P. tolaasii* cells was studied by the two-layer agar method on a solid yeast-peptone medium (DP): yeast extract, 0.3%; peptone (Sigma-Aldrich, St. Louis, MO, USA), 0.06%; diluted 4 times; agar (0.6% and 1.5% for the 1st and 2nd layer, respectively). A total of 5 μL of the predator suspension was applied on the lawn of prey cells, and the results were assessed in the course of 7 days by the formation of lysis zones.

### 2.4. pH Effect

The effect of pH on the growth of *B. bacteriovorus* LR3 in a co-culture with *P. tolaasii* prey cells was assessed in tap water. The culture of *P. tolaasii* was in the late logarithmic phase, with the initial OD_600_ of 1.9 (corresponding to the average initial concentration of prey cells 1.2 × 10^9^ CFU/mL). The pH of tap water (from 2.0 to 9.5) was adjusted with 1% HCl (Sigma-Aldrich, St. Louis, MO, USA) and KOH (Sigma-Aldrich, St. Louis, MO, USA) solutions. The results were assessed by the change in OD_600_ and recorded for 7 days.

### 2.5. Assessing the Spectrum of Lytic Activity

The spectrum of antimicrobial (lytic) activity of the isolated strain was assessed against bacteria that belonged to different taxonomic groups (see Section 3). The lytic spectrum was determined in sterile tap water. A one-day predator culture was filtered three times through 0.45-μm membrane filters. The concentration of LR3 cells after filtration was 10^7^ PFU/mL. The prey bacteria were cultivated on a tryptone–soy medium 5/5. The solid-medium lytic activity was determined in Petri dishes using a two-layer agar method. The predator culture was inoculated into the upper layer of the solid medium (sterile tap water, 0.6% agar) containing test prey bacteria (10^9^ CFU/mL). The lytic activity was estimated by the appearance of a lytic zone around the injection site within 3–5 days after inoculation. The lysis of test bacteria in the liquid medium (sterile tap water; prey cells, 10^7^–10^8^ CFU/mL; predator cells, 10^6^ PFU/mL) was assessed using a spectrophotometer on the 3rd–4th day after inoculation (see, 2.2. Cultivation of the co-culture). The uninfected suspensions of prey cells (10^7^–10^8^ CFU/mL) served as controls. Both solid- and liquid-medium samples were incubated at 30 °C; liquid-medium samples were put on a circular shaker (230 rpm). The antimicrobial effect was considered positive if a lytic reaction was detected by at least one of the methods used. In the course of the experiment, all samples were examined under a microscope.

### 2.6. Light Microscopy

The microscopic examination of culture samples was performed using a Nikon Eclipse Ci microscope (Nikon, Tokyo, Japan) with a phase objective and a ProgRes SpeedXT camera (Jenoptic, Jena, Germany).

### 2.7. Transmission Electron Microscopy (TEM)

The intact cells of the isolated strain were examined as negatively stained (contrasted) preparations. The negative staining was performed using a 0.2% aqueous solution of uranyl acetate (Sigma-Aldrich, St. Louis, MO, USA). The ultrathin sections were prepared as described in [24]. Briefly, bacterial cells were collected by centrifugation, fixed in 2.5% glutaraldehyde (Sigma-Aldrich, St. Louis, MO, USA) for 1 h at 4 °C, and post-fixed with 2% OsO_4_ (Sigma-Aldrich, St. Louis, MO, USA) at room temperature for 12 h. After dehydration in a series of alcohols (70%, 80%, 96% and 100%) and acetone (100%) (Sigma-Aldrich, St. Louis, MO, USA), the samples were immersed in Epon 812 (Sigma-Aldrich, St. Louis, MO, USA) and polymerized sequentially at 37 °C and 60 °C. The ultrathin sections obtained using a Reichert-Jung Ultracut E ultramicrotome (Reichert-Jung, Vienna, Austria) were placed on support grids and contrasted with 3% uranyl acetate in 70% alcohol, followed by additional contrasting with lead citrate according to Reynolds [25]. The ultrathin sections and negatively stained preparations were examined under a JEM-1400 transmission electron microscope (JEOL, Tokyo, Japan) at the accelerating voltage 80 kV.

### 2.8. X-Ray Microanalysis

The elemental composition of the exocellular vesicular ultrastructures was analyzed by X-ray microanalysis on non-contrasted thin sections using a JEM-1400 microscope (JEOL, Tokyo, Japan) equipped with an Oxford Instruments X-ray microanalyzer (Oxford, UK), operating at an accelerating voltage of 80 keV.

### 2.9. Phylogenetic Analysis

DNA extraction, *16S rRNA* gene sequencing, and preliminary phylogenetic analysis were carried out as previously described [24].

### 2.10. Whole Genome Sequencing

DNA of the *Bdellovibrio* strain was isolated from the fresh bacterial biomass using a FastPure Bacterial DNA Isolation Mini Kit (Vazyme, Nanjing, China). Sequencing was performed on SURFSeq 5000 (GeneMind, Shenzhen, China) and DNBSEQ-G50 (MGISEQ-200, MGI, Shenzhen, China) equipment. Information about the number of reads generated is given in Table 1.

MGI reads were unfiltered. SURFSeq reads were filtered using Trimmomatic v. 0.39 [24] with the following parameters: ILLUMINACLIP:TruSeq3-PE-2.fa:2:30:10 LEADING:15 TRAILING:15 SLIDINGWINDOW:4:15 MINLEN:100. Both these datasets were used in assembling the genome de novo.

The bacterial genome was assembled using SPAdes v. 3.15.4 [26]. Short contigs (<500 bp) were removed. The purity of the assembly was tested using CheckM v. 1.2.2 [27]. The test showed that the assembly had a 96.85% completeness and a 0.00% contamination. The ANI value was calculated using the EzBioCloud ANI Calculator (https://www.ezbiocloud.net/tools/ani, accessed on 11 January 2026) [28]. Digital DNA-DNA hybridization (dDDH) was calculated using the Genome-to-Genome Distance Calculator 3.0 (https://ggdc.dsmz.de/ggdc.php, accessed on 11 January 2026) (GGDC) [29]. The genome was annotated with NCBI Prokaryotic Genome Annotation Pipeline (PGAP) version 4.6 [30] and Prokka (https://github.com/tseemann/prokka, accessed on 11 January 2026) [31]. The functional annotation of the genome was carried out using eggNOG mapper 5.0 (http://eggnog5.embl.de/#/app/home, accessed on 11 January 2026).

### 2.11. Statistical Methods

In all of the experiments, samples were made in triplicates. The statistical analysis of the results was performed using software packages SPSS v. 23.0 (IBM, Armonk, NY, USA) and Microsoft Excel 2016 (Microsoft, Redmond, WA, USA). The differences were assessed with one-way ANOVA at the significance level of *p* ≤ 0.05.

## 3. Results

### 3.1. Morphology, Cell Ultrastructure and Development Cycle of B. bacteriovorus LR3

**Figure 1:**
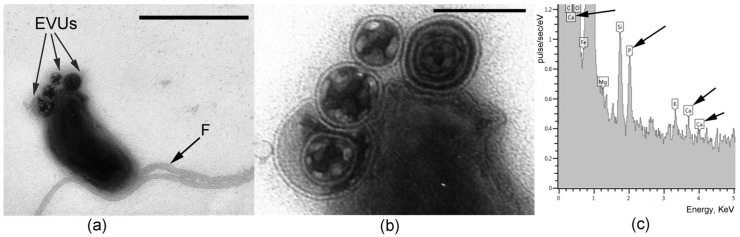
Transmission electron microscopy. (**a**) Morphology of LR3 cells. TEM, negative contrast. The arrows indicate exocellular vesicular ultrastructures (EVUs) and flagellum (F). Scale bar—0.5 µm. (**b**) A fragment of the *B. bacteriovorus* LR3 cell with EVUs on its surface. Scale bar—0.1 µm. (**c**) X-ray spectrum of the EVU content. Arrows point to calcium (Ca) and phosphorus (P) in the EVU composition.

The cells of *B. bacteriovorus* LR3 have a Gram-negative type of cell wall and are shaped as vibrios, 0.5–1.5 μm long, and 0.2–0.3 μm wide (Figure 1a). The LR3 cell has a single polar flagellum, which can reach 5 μm in length (4 times longer than the cell itself).

The electron microscopy examination of negatively stained LR3 cells, which were fixed in the attack stage, revealed the presence of large (0.06–0.16 μm) spherical formations on the cell surface (usually at the poles) and in the intercellular space. These formations are specific exocellular vesicular ultrastructures (EVUs) surrounded by a double membrane. The content of these vesicles is heterogeneous: it consists of a crystalline substance with low electron density (apparently, of protein nature) and electron-dense homogeneous matter (Figure 1b). According to X-ray microanalysis, the vesicle content is rich with P and Ca elements (Figure 1c). Such ultrastructural organization of the EVU matrix is characteristic of the state of *B. bacteriovorus* LR3 antagonistic interaction with all the prey bacteria studied. Presumably, EVUs of the LR3 strain contain a complex of enzymes allowing the predator to penetrate the cell wall of the prey.

Our experiments showed that antagonistic interactions of *B. bacteriovorus* LR3 with most of the Gram-negative bacteria tested were characterized by an infectious cycle typical of *Bdellovibrio* bacteria. The only exception was *P. tolaasii*: with this toxigenic bacterium, a special type of interaction was detected, different in dynamics from the classical scheme.

**Figure 2:**
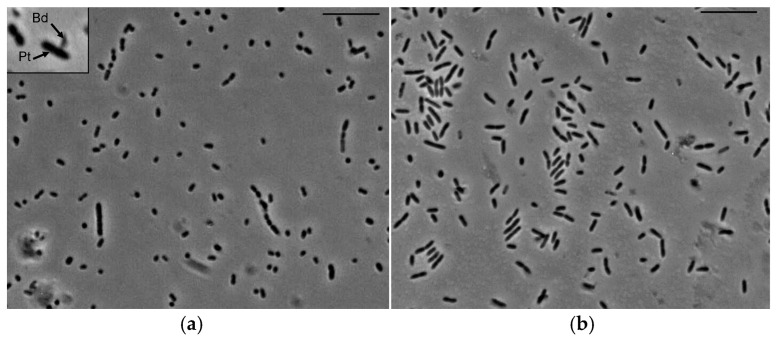
Interaction of *B. bacteriovorus* LR3 with *P. tolaasii*, as revealed by phase-contrast microscopy. Scale bar—10 µm. (**a**) Prey (Pt) and predator (Bd) cells in a co-culture; (**b**) cells of *P. tolaasii* in a one-day monoculture.

Within the first 4 h of LR3 and *P. tolaasii* interaction, no attachment of the predator to the prey was observed. Within the next 8–12 h, however, some of the prey cells seemed to recognize the predator’s presence. As a result, their shape changed: the rod-shaped prey cells became more elongated, which was followed by their fragmentation (Figure 2a). Thus, the number of prey cells in the culture increased. In the control culture (i.e., in the absence of the predator), no such morphological rearrangements of *P. tolaasii* was observed at any of the phases of its growth (Figure 2b).

The morphological rearrangements of *P. tolaasii* cells in response to the LR3 presence were accompanied by changes indicating the beginning of the active phase of the predation process (attachment and penetration of the predator into the prey cells). The infected prey cells were changing their shape from rods to small balls, the so-called bdelloplasts (the intracellular stage of *Bdellovibrio* development).

**Figure 3:**
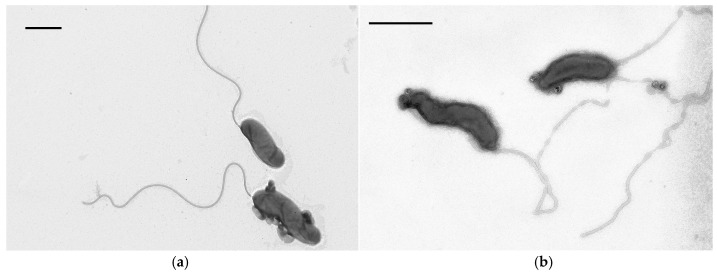
Cells of *B. bacteriovorus* LR3 in a co-culture with *E. coli* (**a**) and *P. tolaasii* (**b**). TEM, negative contrast. Scale bar—0.5 µm.

**Figure 4:**
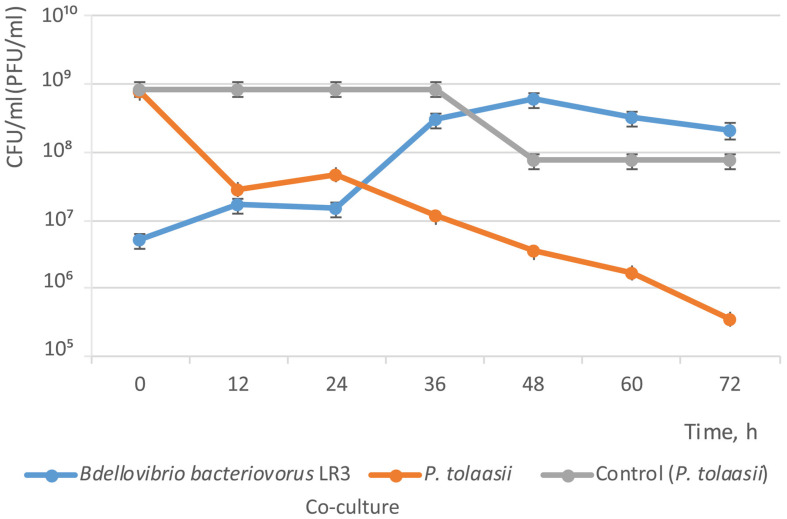
Changes in the number of cells in a co-culture of *B. bacteriovorus* LR3 (PFU/mL) and *P. tolaasii* (CFU/mL). The control was a culture of *P. tolaasii* cells (CFU/mL).

At this stage of interaction between LR3 and *P. tolaasii* cells, LR3 vibrios were gradually disintegrating along the entire length of their flagella into spherical and hemispherical fragments (Figure 3). At the same time, there was no mass release of bdellovibrios from bdelloplasts (the concentration of bdellovibrios at that point did not increase significantly and remained at a level of 10^7^ PFU/mL). By this moment, the concentration of *P. tolaasii* cells decreased by an order of magnitude and was about 10^7^ CFU/mL. Within the next 2 days, the concentration of bdellovibrios increased to 10^8^ PFU/mL, and the concentration of prey cells decreased to 10^5^ CFU/mL (Figure 4). According to the data of light microscopy, the increase in the number of lytic spots was mainly related to bdelloplasts (intracellular bdellovibrios).

**Figure 5:**
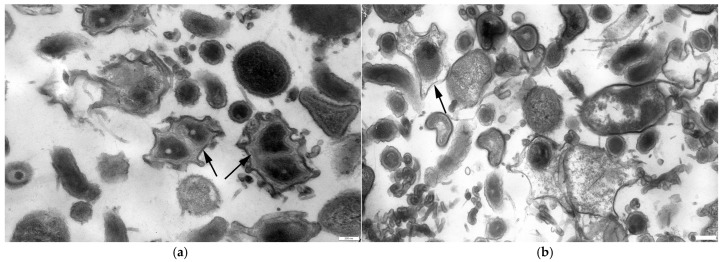
Formation of bdelloplasts (arrow) in the process of interaction of *B. bacteriovorus* LR3 with *P. tolaasii* (**a**) and *E. coli* (**b**). TEM, ultrathin sections. The scale bars are equal to 200 nm.

During the interaction phase (3 days), bdelloplasts in the co-culture suspension adhered to each other, forming conglomerates. The formation of conglomerates was observed under all of the experimental conditions studied, so their presence, although affecting the optical density of the media, did not interfere with the comparative analysis of the samples. On the first day, these conglomerates were small clusters, which could disintegrate as bdellovibrios were released from the prey cells (Figure 5a). By the third day, large clusters of bdelloplasts had formed, and the release of bdellovibrios from bdelloplast became insignificant. Surprisingly, with the majority of the predator population in the bdelloplast state, the active lysis of *P. tolaasii* cells continued. In the control cultures, *P. tolaasii* cells were slightly lysed, and within 72 h, their number decreased by an order of magnitude.

In the co-cultures with other LR3-sensitive bacteria, the studied strain followed the classic pattern of *Bdellovibrio* predation. For example, its attachment to the cells of *E. coli* C600 was observed after 4 h of cultivation, with the formation of bdelloplasts registered after 5–6 h (Figure 5b). No disintegration of flagella in LR3 cells was observed in these cases.

### 3.2. Phylogenetic Position of the Strain LR3

**Figure 6:**
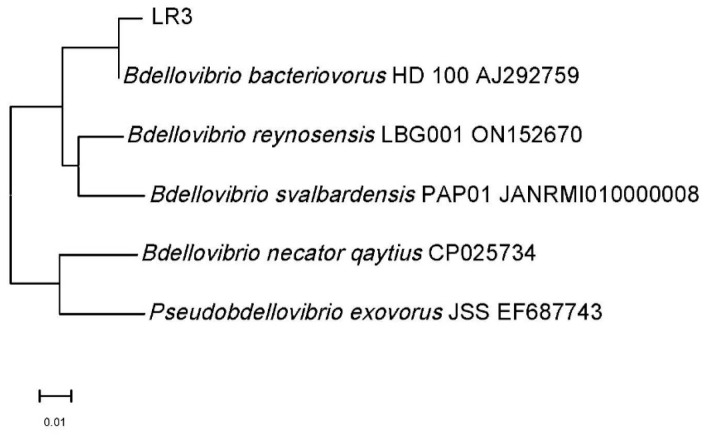
A phylogenetic tree based on the analysis of the gene sequence of 16S rRNA. The tree shows the position of the LR3 strain in relation to other representatives of the genus *Bdellovibrio*.

The isolated strain of predatory bacteria was identified by 16S rRNA analysis as *Bdellovibrio bacteriovorus* LR3. Its identity with *Bdellovibrio bacteriovorus* HD 100 was 99% (Figure 6).

### 3.3. General Characteristics of the Genome and Strain Identification

The genome contained 3611 genes in total, of which 3568 were coding sequences (CDSs). Out of 3568 CDSs, 3562 were CDSs with protein and 6 CDSs without protein (pseudogenes). The genome contained 43 RNA genes in total, of which 35 were tRNAs, 4 ncRNAs, and 4 rRNAs (a full (5S, 16S, 23S) and partial (23S) clusters of ribosomal RNA). The genome data can be accessed in the GenBank database under the accession number JBNOXV010000000 (BioProject PRJNA1258711, BioSample SAMN48328637).

The reference strain used to identify LR3 was a type *Bdellovibrio* strain *B. bacteriovorus* HD100 (assembly number GCF_000196175.1, BioProject PRJNA224116). The HD100 genome consists of a single circular chromosome 3,782,950 bp in length. The metrics of the LR3 genome assembly are given in Table 2.

The calculation of genomic indices clearly indicates that LR3 belongs to the *B. bacteriovorus* species. The ANI (orthoANIu) and dDDH values of LR3 vs. *B. bacteriovorus* HD100^T^ are 98.59% and 96.8%, respectively.

The analysis of the annotated genome sequence of *B. bacteriovorus* LR3 revealed a number of genes, which could be involved in the formation of the functional potential of the strain, in particular:

1. Genes for the production of secondary metabolites. The clusters of terpene precursor synthesis are located on the contig Node 1, at the coordinates 1,806,505–1,827,461 bp (total length: 20,957 bp) and 2,141,892–2,162,761 bp (total length: 20,870 bp). The genes for the synthesis of type III polyketide synthase (T3PKS) are located at the coordinates 1,996,069–2,037,178 bp (total length: 41,110 bp). The genes for terpene synthesis are located on the contig Node 2, at the coordinates 674,265–695,557 bp (total length: 21,293 bp). The siderophore synthesis cluster is located on the contig Node 2 at the coordinates 157,875–190,348 bp (total length: 32,474 bp).
2. Genes involved in the metabolism of peptidoglycan. These genes are involved in the development of *B. bacteriovorus* cells, providing for the functionality that relates to bacterial parasitism and predation. The Glycan metabolism category is represented by 74 genes, which can be further divided into the following subcategories: Amino sugar and nucleotide sugar metabolism (14 genes); Biosynthesis of various nucleotide sugars (22 genes); N-Glycan biosynthesis (1); Glycosaminoglycan degradation (1); Lipopolysaccharide biosynthesis (11); Peptidoglycan biosynthesis (21); Teichoic acid biosynthesis (2); Exopolysaccharide biosynthesis (2).

### 3.4. Effects of Temperature and pH on the Interaction of B. bacteriovorus LR3 with P. tolaasii

The interactions in the two-component system of *B. bacteriovorus* LR3 and *P. tolaasii*, which affect the harvest yield of *A. bisporus*, largely depends on the cultivation temperature. In this work, we tested the effect of temperature (5–45 °C) on the growth of the bacterial co-culture on a solid medium and found that the predator–prey interactions were most active in the range of 10–37 °C. At the extreme temperatures (5 and 45 °C), no lytic zones were detected. At 20, 25, and 30 °C, lytic spots appeared on the 3rd day of incubation; at 10, 35, and 37 °C, on the 4th–5th day. On the basis of these results, the temperature of 30 °C was chosen for further experiments.

**Figure 7:**
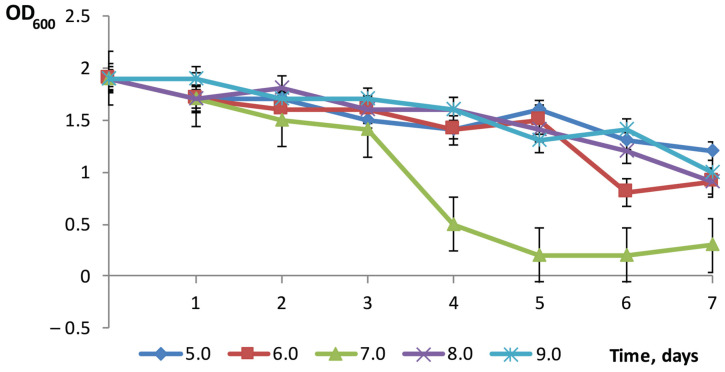
Dynamics of the predator–prey interaction in the system of *B. bacteriovorus* LR3 and *P. tolaasii* depending on the pH of the medium.

Another important factor, affecting for interactions in the predator–prey system, is pH. Our experiments showed that lysis of *P. tolaasii* cells in the liquid predator–prey co-culture occurred in the pH range 5.5–7.5 (Figure 7). The lysis, which was assessed by the clarification of the medium, was maximal at neutral pH. On the 4th–5th days of cultivation, the pH of all of the cultivated samples changed towards neutral values.

### 3.5. Spectrum of Antimicrobial (Lytic) Activity of B. bacteriovorus LR3

Our tests showed that the LR3 strain was capable of lysing a broad spectrum of Gram-negative bacteria and, at the same time, did not interact with Gram-positive microorganisms (Table 3). LR3 was especially active towards *Escherichia coli* (all the strains tested), *Salmonella enteridicum* R6, *Alcaligenes faecalis* VKM B-1518, and a number of *Pseudomonas* strains, being most effective against *P. aeruginosa* ML4262 and *P. tolaasii*.

**Table 3:** Antimicrobial activity of *B. bacteriovorus* LR3.

No.	Prey Bacteria	Presence (+) or Absence (−) of Lysis	
		In Liquid Medium	On Solid Medium
	Gram-Negative Bacteria		
1	*Aeromonas veronii* *	−	−
2	*Alcaligenes faecalis* VKM B-1518	+	+
3	*Alcaligenes ruhlandii* VKM B-1333	−	−
4	*Escherichia coli* 5 K *	+	+
5	*Escherichia coli* B 102 *	+	+
6	*Escherichia coli* C 600 *	+	+
7	*Escherichia coli* S *	+	+
8	*Erwinia carotovora* VKM B-15	−	+
9	*Erwinia herbicola* ATCC 27155	−	+
10	*Pseudomonas aeruginosa* PAO1 *	+	−
11	*Pseudomonas aeruginosa* ML4262 *	+	+
12	*Pseudomonas chlororaphis* OV17 *	−	+
13	*Pseudomonas fluorescens* VKM B-894	+	−
14	*Pseudomonas putida* VKM B-149	−	−
15	Pseudomonas tolaasii *	+	+
16	*Salmonella enteritis* R6 *	+	+
	Gram-Positive Bacteria		
17	*Bacillus subtilis* ATCC 6633	−	−
18	*Deinococcus radiodurans* VKM B-1422	−	−
19	*Micrococcus luteus* VKM B-1891	−	−
20	*Staphylococcus* sp. St. 35 *	−	−

VKM in the name of a bacterial strain means that it was provided by the All-Russian Collection of Microorganisms (IBPM, PSCBR RAS, Pushchino, Russia); ATCC, that the strain was from the American Type Culture Collection (Manassas, VA, USA). * The strains are from the collection of the Laboratory of Cytology of Microorganisms (IBPM PSCBR RAS, Pushchino, Russia).

## 4. Discussion

In this work, we isolated and characterized a new strain of *Bdellovibrio* bacteria. The strain had been isolated from water of a pond near the dam of the Lyubozhikha River and cytologically characterized as a periplasmic predator. On the basis of the analysis of its 16S rRNA gene sequence, it was classified as *Bdellovibrio bacteriovorus* LR3. As demonstrated by our experiments, the LR3 strain preys upon a wide range of Gram-negative bacteria and is particularly effective against *Pseudomonas tolaasii*, a toxicogenic bacterium infecting *Agaricus bisporus* [7].

The interaction of *B. bacteriovorus* LR3 with *P. tolaasii* differs from the classical pattern of *Bdellovibrio* predation—both in dynamics and behavior. The differences are related to, on the one hand, the prey’s defensive strategies and, on the other hand, predator’s methods aimed at overcoming these strategies.

The dynamical differences are particularly related to the onset and duration of the attack phase. The formation of bdelloplasts in the co-culture of *B. bacteriovorus* LR3 and *P. tolaasii* occurred 8–10 h after their interaction, which is a much longer period in comparison with the usual 2–3 h of the classical pattern. Overall, this leads to the substantial prolongation of the predation cycle. As demonstrated in our experiments, the stage of intracellular growth of LR3 cells in the co-culture with *E. coli* C600 (until the release of the predator cells from bdelloplasts) was completed in 36–48 h, whereas in the system with *P. tolaasii*, it took about 72 h. The delay of the attachment stage in the LR3 predation cycle is probably caused by the presence of tolaasin in the medium, which can immobilize *Bdellovibrio* cells. This hypothesis requires further investigation into the effect of tolaasin on the Bdellovibrio life cycle, including through the use of compounds that inhibit the toxin’s activity [32].

Moreover, sometimes the rod-shaped cells of the toxigenic bacteria divide into smaller cells, thereby increasing the size of the *P. tolaasii* population. This indicates that the prey has detected the presence of the predator and triggered a survival response at the population level (with the prey cells exceeding the predator ones in numbers, a part of the *P. tolaasii* population may survive).

To deal with the poisonous defense of *P. tolaasii*, *B. bacteriovorus* LR3 implements its own strategy of “fortifying and waiting” in the bdelloplast state. In our earlier work [33], we demonstrated that the cell membrane of bdelloplasts functions as a protective barrier: after penetrating into the periplasm of the victim cell, a bdellovibrio can tolerate both adverse environmental pressures and starvation (if the prey population is thinned). The intracellular stage seems to be predominant in the development cycle of *Bdellovibrio*; lingering in this stage is, most probably, a survival mechanism.

When cultivated with *P. tolaasii*, the LR3 *Bdellovibrio* strain further enhances the “bdelloplast fortification” strategy. As revealed by light microscopy, bdelloplasts in the LR3–*P. tolaasi* system form a large number of conglomerates. By the end of the intracellular growth stage, the number of conglomerates decreases, but they grow in size. One possible advantage of conglomerate formation is that it helps to optimize the local environmental conditions. It is known that *Bdellovibrio* is sensitive to adverse environmental pressures during the extracellular non-reproductive stage of its development. For example, the mobility of bdellovibrios decreases at low pH [34]. Our experiments have shown that *B. bacteriovorus* LR3 is active towards *P. tolaasii* (i.e., capable of clarifying the culture suspension within 7 days) in the range of pH 6.0–8.0. The pH optimum of the predatory activity (corresponding to the maximal clarification of the co-culture) lies in the range of 6.5–7.5 (Figure 7). However, even at optimal pH, clarification of the co-culture only occurs on the 4th day of incubation—and this is under the conditions when almost all of the predator’s cells are already in the state of bdelloplasts.

Conglomeration of bdelloplasts may also facilitate another important task: neutralization of tolaasin in the vicinity of the predator cells. It is easier to detoxicate the inner space of conglomerates if the diffusion of the toxin from the outer space is restricted.

Thus, prolongation of the predatory cycle of *B. bacteriovorus* LR3 in its antagonistic interaction with *P. tolaasii* is underlain by the extension of the pre-attack stage. After the initial predator–prey recognition, there is a clash of adversarial prey and predator strategies, with the fight centered around the specific toxin released by *P. tolaasii*, tolaasin. According to our observations, *P. tolaasii* uses tolaasin for both attacking *A. bisporus* and protecting itself from *B. bacteriovorus* LR3. The toxin causes the predator to slow down its onslaught and spend some time to adapt to/neutralize the poison before finding new cells to prey upon. Apparently, the LR3 strain has found a way to survive tolaasin poisoning; however, further studies are needed to elucidate the mechanisms, by which LR3 cells protect themselves during the extracellular stage of the predation cycle. In any case, tolaasin can only slow down but not completely inhibit the process of antagonistic interaction of *B. bacteriovorus* LR3 and *P. tolaasii.*

The slow-paced predatory behavior of *B. bacteriovorus* LR3 poses a question, which is both theoretically interesting and practically important: how does the predator exert its antimicrobial effect from the bdelloplast state? One can assume that the lytic activity of *B. bacteriovorus* LR3 is mediated by the enzyme-loaded exocellular vesicles produced by LR3 cells in large numbers at the beginning of the predator–prey interaction. It is also possible that being ruptured by the LR3 lytic enzymes, the membrane of *P. tolaasii* cells becomes permeable to their own toxin secreted in the outer medium. In our future studies, we are planning to examine the possibility that during its prolonged bdelloplast stage, *B. bacteriovorus* LR3 not only protects itself from the effect of adverse environmental factors, but also puts pressure on the cells of *P. tolaasii* through a vesicle-mediated mechanism.

The first data, describing interaction of *Bdellovibrio* bacteria (*B. bacteriovorus* HD100) with *P. tolaasii*, were published in [21], with the work also discussing the possibility of using bdellovibrios to treat harvested mushrooms of *A. bisporus* from brown spot. Both bacteria (the prey and the predator) are natural inhabitants of the upper layer of soil, which is also the place where the mycelium of *A. bisporus* grows [8]. *Bdellovibrio* bacteria can move with record speed in the aquatic environment [35] and are also capable of hunting their prey on solid surfaces [36]. When adhered to a surface, bdellovibrios slowly glide [37], using chemotaxis to locate areas rich in prey [38]. Be it in vitro or in vivo, they are well-equipped for hunting *P. tolaasii*, and their introduction into the casing soil can prevent the infection of *A. bisporus* by phytopathogenic pseudomonads, slowing down the spread of brown spot at the stage of mushroom formation.

In fungiculture, the use of antimicrobial agents with broad specificity (chemicals, antibiotics and predatory bacteria that indiscriminately hunt a wide spectrum of preys) is limited. Treating the soil with such agents can suppress the growth of useful bacteria, such as *P. putida*, which is necessary to promote the development of mushroom bodies at the initial stages of their formation [20,21,22]. Correspondingly, after isolation and purification of a new *Bdellovibrio* strain, we identified the spectrum of its antimicrobial activity (Table 3). One can see that the isolated strain has a broad spectrum of action against Gram-negative bacteria, does not interact with Gram-positive microorganisms—but most importantly, is not active towards *P. putida* VKM B-149. Hence, we can conclude that *B. bacteriovorus* LR3 can be used in *A. bisporus* farming as an antimicrobial agent suitable for the treatment of soil at the stage of development of mushroom bodies.

The analysis of the lytic activity of *B. bacteriovorus* LR3 in solid and in liquid cultures has also shown that among all of the prey bacteria tested, the new strain was most effective against *Escherichia coli* C600, *E. coli* B, *E. coli* S, *E. coli* 5K, *Salmonella enteriditis* R6, *Alcaligenes faecalis* VKM B-1518, *Psseudomonas aeruginosa* ML4262, and *P. tolaasii* (Table 3). In some cases, the antimicrobial activity of *B. bacteriovorus* LR3 was even strain-specific: two different strains of *P. aeruginosa* were lysed by LR3 to a different degree. Such a specificity can even be compared with that of bacteriophages.

The effectiveness and broad specificity of *B. bacteriovorus* LR3 against *E. coli* suggests that it is a prospective candidate for future study as an agent against APEC strains [39,40]. Furthermore, *B. bacteriovorus* LR3 is able to lyse *Salmonella enteriditis* R6, a representative of the *enteriditis* serovar, which is a major source of eggs/poultry meat-related foodborne infections in humans [41,42]. However, the research experience in this area is limited [43], which also supports the idea of testing the ability of *B. bacteriovorus* LR3 to lyse the causative agents of enterobacterioses in broiler chickens.

Thus, the new LR3 strain of the predatory bacterium *Bdellovibrio bacteriovorus* seems promising for use in farming (fungiculture, poultry) and medicine. The ability of the LR3 strain to hunt its prey at 25–37 °C might also be used for in situ biocontrol tests in application to phytopathogenic and zoonotic Gram-negative bacteria.

## 5. Conclusions

In this study, we isolated and characterized a predatory bacterium, *Bdellovibrio bacteriovorus* LR3, from the Lyubozhikha River. This strain was identified as a periplasmic predator capable of lysing a broad spectrum of Gram-negative microorganisms, including plant pathogens. Specifically, LR3 exhibited pronounced antagonistic activity against *Pseudomonas tolaasii*, the causal agent of brown spot on *Agaricus bisporus*. Importantly, LR3 remained active across the temperature and pH ranges typical for *Agaricus bisporus* cultivation and did not affect *Pseudomonas putida*, a beneficial species essential for mushroom fruiting. This selectivity indicates that LR3 is a promising biological control agent in mushroom cultivation. Beyond its agricultural applications, LR3 demonstrated strong lytic activity against multiple strains of *Escherichia coli* and *Salmonella enteriditis*, suggesting its potential for use in poultry farming and related biomedical research. Overall, LR3 *B. bacteriovorus* combines ecological adaptability with selective and effective predation, confirming its potential as an effective microbial control agent and suggesting further in vivo experiments.

## Figures and Tables

**Table 1:** Preparation of libraries and the number of generated paired-end reads.

Equipment	SURFSeq 5000	DNBSEQ-G50
Length of reads, bp	150	100
Number of generated reads	2,061,705	3,861,159

**Table 2:** Metrics of the LR3 genome assembly.

Parameter	Value
Number of contigs	11
Genome size, bp	3,824,270
N50, bp	2,936,311
Longest contig, bp	2,936,311
Shortest contig, bp	1051
N75, bp	2,936,311
N90, bp	873,810

## Data Availability

The original contributions presented in this study are included in the article. Further inquiries can be directed to the corresponding author.
